# Whistle-blowers – morally courageous actors in health care?

**DOI:** 10.1177/09697330221092341

**Published:** 2022-06-21

**Authors:** Johanna Wiisak, Riitta Suhonen, Helena Leino-Kilpi

**Affiliations:** 8058 University of Turku, Department of Nursing Science, Turku, Finland; University of Turku, Department of Nursing Science and Director of Nursing, 60652 Turku University Hospital, and City of Turku, Welfare Services Division, Finland; University of Turku, Department of Nursing Science and Director of Nursing, 60652 Turku University Hospital, Finland

**Keywords:** Whistleblowing, whistle-blower, moral courage, nurse, health care, video vignette method

## Abstract

**Background:**

Moral courage means courage to act according to individual’s own ethical values and principles despite the risk of negative consequences for them. Research about the moral courage of whistle-blowers in health care is scarce, although whistleblowing involves a significant risk for the whistle-blower.

**Objective:**

To analyse the moral courage of potential whistle-blowers and its association with their background variables in health care.

**Research design:**

Was a descriptive-correlational study using a questionnaire, containing Nurses Moral Courage Scale^©^, a video vignette of the wrongdoing situation with an open question about the vignette, and several background variables. Data were analysed statistically and inductive content analysis was used for the narratives.

**Participants and research context:**

Nurses as healthcare professionals (including registered nurses, public health nurses, midwives, and nurse paramedics) were recruited from the membership register of the Nurses’ Association via email in 2019. A total of 454 nurses responded. The research context was simulated using a vignette.

**Ethical considerations:**

Good scientific inquiry guidelines were followed. Permission to use the Nurses’ Moral Courage Scale^©^ was obtained from the copyright holder. The ethical approval and permission to conduct the study were obtained from the participating university and the Nurses’ Association.

**Findings:**

The mean value of potential whistle-blowers’ moral courage on a Visual Analogue Scale (0–10) was 8.55 and the mean score was 4.34 on a 5-point Likert scale. Potential whistle-blowers’ moral courage was associated with their socio-demographics, education, work, personality and social responsibility related background variables.

**Discussion and conclusion:**

In health care, potential whistle-blowers seem to be quite morally courageous actors. The results offer opportunities for developing interventions, practices and education to support and encourage healthcare professionals in their whistleblowing. Research is needed for developing a theoretical construction to eventually increase whistleblowing and decrease and prevent wrongdoing.

## Introduction

Even though whistleblowing has been studied for decades, there is a growing interest towards it among researchers.^1,2^ Whistleblowing is an individual’s response to wrongdoing, such as unsafe care, inappropriate or unethical behaviour or illegal activity.^3–7^ Even though whistleblowing is usually done with good intentions for ending the wrongdoing, the reactions towards the whistle-blower vary from appreciation to retaliation.^4,6,7^ Therefore, whistleblowing still holds its place as a current and problematic issue in society globally^8,9.^ Virtue ethics has been proposed as a foundation for whistleblowing^10,11^ and courage as one of the fundamental human virtues,^12–14^ acknowledged also in health care and nursing.^15–17^ This study focuses on nurses as potential whistle-blowers and their moral courage, as they represent the largest group of healthcare professionals.

## Background

In this study, whistleblowing is defined as a three-phased process where: (1) wrongdoing such as malpractice, workplace bullying or stealing medicine^4,6,7^ is suspected or observed in health care by a healthcare professional. Healthcare professionals become whistle-blowers if they (2) address the whistleblowing act inside the organisation to a person or party capable of ending the wrongdoing such as managers or outside to police, health authorities or media. Whistle-blower may suffer (3) consequences such as retaliation or discrimination after performing the whistleblowing act.^6,7,18,19^ Within this definition, the whistleblowing act is considered to include internal whistleblowing acts as speaking up or raising concerns to the wrongdoer, colleagues or the closest manager.^9,20^ Whistle-blowers are generally seen as brave as they act despite potential risks for themselves,^8,21^ such as retribution or ostracism, after performing the act of whistleblowing.^2–7,22–24^ The threat of negative consequences is prevailing, even when the act of whistleblowing is addressed anonymously.^25,26^ Whistle-blowers may repeatedly perform whistleblowing acts which are ignored and turn into ineffective efforts to end the wrongdoing.^21^ Even when a healthcare professional is aware of a morally appropriate action required when observing wrongdoing, one could be unable to act due to organisational or personal obstacles such as punitive workplace culture or lack of courage.^7,27^ Particularly in health care context, these increase healthcare professionals’ moral distress and may eventually lead to turnover or leaving the profession,^16,28^ thereby worsening an already poor work force situation and shortage of professionals.^29^ Moral courage is considered as an empowering and positive way to reduce moral distress.^17,27^

Moral courage is value-oriented, determined through individual -value base. It can be considered as an individual’s inner strength and as an appreciated human virtue. Moral courage is said to be part of an individual’s ethical competence^14,30^ that can be strengthened and developed^31^ through ethics education, educational interventions, and ethical discussions.^13,32,33^ Moral courage is the ability to overcome fears of the potential negative consequences to oneself and to defend professional and personal ethical principles and values. It is about having an inner will to do the right thing, for the good of others. In addition, expressing moral courage means acting without external force by sacrificing oneself and still feeling a sense of peace. Moral courage is “the golden mean” between cowardice and rashness, with the former indicating irrational fear and the latter thoughtlessness.^16,31,33,34^ In the health care context, research about moral courage has been scarce, although it has increased during the past decade, particularly among nurses^33,35–37^ and nurse students.^38–41^ The characteristics of a morally courageous nurse^14,30,32,33,37,38,42^ and the background variables associated with moral courage have been described in previous literature.^36,41^ Morally courageous nurses are ethically sensitive, willing to risk themselves for the benefit of others and to act according to their own conscience.^37,43–45^ They feel responsibility for others^14,32,45^ and possess confidence in situations requiring moral courage^14,43^ and are confident in their own ethical principles^41^ and value base.^37^

Studies describe associations between moral courage and socio-demographics: age^41^ and gender.^36^ However, some studies indicate that in terms of moral courage, gender is not a significant variable.^32,45^ In addition, education related variables – additional ethics education, continuing education,^32,37,46^ higher level of education and previous degree in health care^41^ – are associated with moral courage and beneficial for strengthening it.^32^ Among work-related variables as working in management position^36^ and having longer work experience^14,41^ are associated with moral courage. Moreover, associations were described between moral courage and the frequency of encountering situations requiring moral courage at work,^36^ having nursing career plans as well as dissatisfaction with nursing profession.^41^

Moral courage is expected to increase the probability of whistleblowing,^47^ and differences in the level of moral courage could explain why some individuals are willing to become whistle-blowers while others are not.^13,47,48^ However, there appears to be a gap in the empirical research about whistle-blowers and their moral courage in health care. Thus, identifying whistle-blowers and their moral courage provides evidence-based knowledge that potentially enhances our understanding of whistle-blowers’ moral courage and how to support and encourage healthcare professionals in their whistleblowing.

### The aim of the study

The aim of this study was to analyse the moral courage of potential whistle-blowers (PW-Bs) and its association with their background variables in health care. The goal of the study was to identify PW-Bs to support and encourage healthcare professionals in their whistleblowing.

The following research questions were addressed:

1. Who are the potential whistle-blowers in health care?
2. What is, the potential whistle-blowers’ self-assessed level of moral courage?
3. What potential whistle-blowers’ background variables, if any, are associated with their self-assessed moral courage?

## Methods

### Design

The research design was a descriptive-correlational study using a questionnaire, containing Nurses Moral Courage Scale^©^ (NMSC), a video vignette of the wrongdoing situation with an open question about the vignette, and several background variables. A scripted video vignette was used as the whistleblowing phenomenon is challenging to capture in real-life. Additionally, video vignettes have been acknowledged as a suitable research method for exploring individuals’ decision-making in situations such as whistleblowing. They enable participants to respond to the same wrongdoing, in the same context, under the same conditions, thereby decreasing situational factors and allowing researchers to focus on individual-related variables.^49,50^

### Participants and data collection

A random sampling on national level was used. Data were collected between 16 August and 5 September 2019 from the membership register of the Nurses’ Association (including registered nurses, public health nurses, midwives, and nurse paramedics). An email containing an invitation to participate in the study and a link to an electronic questionnaire was sent to 30,000 nurses, in the membership register, by the membership coordinator of the association. Based on statistical power analysis^51^ with a significance level of *p* < 0.05 and 95% confidence level, the sample size estimation was 380 nurses.

Altogether 1461 participants returned the questionnaire. Of them, 706 provided a narrative in response to an open question about the video vignette. However, only 454 participants were included in this study, as they described both (1) observing the wrongdoing in the video vignette, and (2) acting as blowing the whistle, in their narratives, giving a response rate of 31%. The participants were working in various health care organisations across the country and representative of an average nurse on a national level.

### Questionnaire

The questionnaire for data collection comprised three parts. Firstly, nurses’ self-assessed level of moral courage was measured using the NMCS^©^.^33^ The development and validation studies show good reliability, validity, and internal consistency for the NMCS^©^.^33,34^ The NMCS^©^ consists of 21 items measuring nurses’ moral courage within four dimensions: 1) *compassion and true presence* (5 items), 2) *moral responsibility* (4 items), 3) *moral integrity* (7 items), and 4) *commitment to good care* (5 items). The responses are given on a 5-point Likert-scale between 1 = “Does not describe me at all” to 5 = “Describes me very well”. Lower scores indicate lower self-assessed level of moral courage and vice versa. In addition, one question measured nurses’ overall moral courage using a Visual Analogue Scale (VAS 1–10) where 1 = “I never act morally courageously even though the care situation would require it” and 10 = “I always act morally courageously when the care situation requires it”.^33,34^

The dimensions In NMCS© are based on a concept analysis.^30^ The dimension of *“Compassion and true presence”* requires courage on the part of the nurse courage to overcome their own fears and vulnerability to encounter the suffering and vulnerability of the patient. *“Moral responsibility”* means taking responsibility and acting courageously in situations where wrongdoing occurs despite the possible powerlessness caused by internal or external circumstances such as hierarchy. *“Moral integrity”* focuses on nurse’s adherence to the ethical principles and values of the profession in situations where there is a risk for potential negative consequences. *“Commitment to good care”* focuses on situations where good care is under threat due to poor care or wrongdoing, for example. The nurse needs courage to advocate for the patient and defend the moral goal of nursing, the patient’s ultimate good.^30,33^

Secondly, the questionnaire included a video vignette of a wrongdoing situation and an open question about the wrongdoing visible in the vignette: “How would you act in the situation and why?” The video vignette took place in home care. In the video vignette, two nurses are visiting an elderly patient with memory disorder, and while the other nurse is measuring the patient’s blood pressure and blood sugar, the other nurse distributes the patient’s medicines and puts a package of medicine into her own pocket. The participants were instructed and able to watch the video once. Lastly, several background variables were asked: (1) socio-demographics, (2) education, (3) work, (4) personality, and (5) social responsibility related variables (Table 1), potential whistleblowing acts, and the ease of acting morally courageously in different ways and confronting different persons or the organisation (Table 2).

**Table 1.** Potential whistle-blowers’ background variables.

Background variables	N	Mean	SD.	Range	f (%)
Socio-demographics	—	—	—	—	—
Age (years)	451	47.0	11.2	21–77	—
Gender	449	—	—	—	—
Female	—	—	—	—	428 (95)
Male	—	—	—	—	21 (5)
Education related variables	—	—	—	—	—
Degree of the profession	432	—	—	—	—
Registered nurse	—	—	—	—	389 (90)
Public health nurse, midwife, nurse paramedic	—	—	—	—	22 (5)
Other (e.g. specially trained nurse, master of administrative sciences)	—	—	—	—	21 (5)
Highest degree	447	—	—	—	—
Student	—	—	—	—	11 (3)
Vocational school degree	—	—	—	—	134 (30)
Polytechnic degree	—	—	—	—	238 (53)
Bachelor or master of health care or health sciences, doctor of health sciences	—	—	—	—	64 (14)
Work related variables	—	—	—	—	—
Work experience (years)	447	19.8	11.5	0–49	—
Present work role	451	—	—	—	—
Employee (registered nurse, public health nurse, midwife or nurse paramedic)	—	—	—	—	284 (63)
Health care manager	—	—	—	—	59 (13)
Not working at the moment	—	—	—	—	43 (10)
Other (e.g. nurse expert, lecturer, practical nurse)	—	—	—	—	65 (14)
Frequency of utilizing ethical or collegial guidelines	451	—	—	—	—
Never	—	—	—	—	21 (5)
Seldom	—	—	—	—	80 (18)
Sometimes	—	—	—	—	112 (25)
Quite often	—	—	—	—	158 (34)
Very often	—	—	—	—	80 (18)
Frequency of situations needing moral courage	453	—	—	—	—
Never	—	—	—	—	0
Seldom	—	—	—	—	16 (4)
Sometimes	—	—	—	—	241 (52)
Quite often	—	—	—	—	166 (37)
Very often	—	—	—	—	30 (7)
Personality related variables	—	—	—	—	—
Personality type	452	—	—	—	—
Introvert	—	—	—	—	73 (16)
Extrovert	—	—	—	—	155 (34)
Between introvert and extrovert	—	—	—	—	224 (50)
Respecting one’s own profession	451	85.2	15.1	18–100	—
Self-esteem	447	73.3	19.0	16–100	—
Allowing others to influence one’s own opinions	441	44.8	18.9	5–90	—
Internal locus of control	443	58.5	16.9	2–100	—
Social responsibility related variables (level of)	—	—	—	—	—
Responsibility for patients or their next of kins	451	84.6	12.9	25–100	—
Responsibility for co-workers	443	75.2	16.7	3–100	—
Responsibility for the well-being of the work community	444	81.2	13.4	29–100	—

**Table 2.** Easiness to act morally courageously.

Acting morally courageously	n	Mean	SD.	Range
Behaviour with moral courage when facing different persons or organization (how easy it is to act)	—	—	—	—
Co-worker (e.g. other nursing professional)	435	4.25	0.80	1–5
Colleague	436	4.14	0.83	1–5
Health care manager (e.g. ward manager)	435	3.82	1.05	1–5
Physician	434	3.53	1.11	1–5
Organization (e.g. executive nurse, administration)	434	3.35	1.20	1–5
Party outside the organization (e.g. authorities)	433	3.62	1.11	1–5
Patient	343	3.99	0.89	1–5
Patient’s next of kin	435	3.83	1.01	1–5
Acting morally courageously in different ways (how easy it is)	—	—	—	—
Bringing the problem into discussion	431	4.16	0.83	1–5
Filing a notification with the organization	430	3.73	1.07	1–5
External whistleblowing (e.g. authorities/trade union/media)	429	3.11	1.16	1–5
Acting morally courageously other way (e.g. discussing with the patient or their next of kin)	306	3.09	0	1–5

### Data analysis

The data were analysed statistically^52^ and the narratives were analysed according to the steps of inductive analysis technique.^53^ The narratives were analysed to identify the participants who described observing the wrongdoing in the video vignette and their potential whistleblowing acts regarding it. Descriptive statistics (frequencies, percentages, mean values and standard deviations) were used to describe the participants’ background variables (Table 1), potential whistleblowing acts (Table 2), participants’ moral courage (Table 3) and easiness of acting morally courageously (Table 4). The following variables were combined to equalise the amounts of responses between the groups: the professional degree and the highest degree. The associations between moral courage and background variables were analysed using Mann-Whitney U-test and Kruskal–Wallis test as the distribution of the data was asymmetric. For the same reason, correlations were examined with Spearman’s correlations. *p* value below 0.05 was considered statistically significant. The data analysis was conducted using R version 4.0.2 software (The R Foundation for Statistical Computing, Vienna, Austria).

**Table 3.** Potential whistle-blowers’ self-assessed moral courage.

Moral courage	n	Mean	SD.	Range
NMCS© total	419	4.34	0.47	2.75–5
Compassion and true presence	437	4.55	0.46	3–5
Moral responsibility	438	4.24	0.58	2.25–5
Moral integrity	430	4.36	0.5	2.71–5
Commitment to good care	436	4.21	0.56	2.8–5
Overall moral courage	439	8.55	0.84	6–10

**Table 4.** Background variables associated with moral courage and its dimensions.

Background variable	Total moral courage *p*-value	Compassion and true presence *p*-value	Moral responsibility *p*-value	Moral integrity *p*-value	Commitment to good care *p*-value	Overall moral courage *p*-value
The degree of the profession	—	—	—	0.036*	—	—
The highest degree	—	—	0.026*	—	—	0.032*
Present work role	<0.001**	<0.01**	<0.001**	<0.001**	<0.001**	0.005**
Personality type	<0.001**	0.006**	<0.001**	<0.001**	<0.001**	<0.001**

Statistically significant *p*-values are indicated in the table: *p < 0.05; **p < 0.01.

## Ethical considerations

This study was conducted following good scientific standards^54,55^ and publication ethics.^56^ Ethical approval was obtained from the Ethics Committee of the University (10/2019) and other permissions from the Nurses’ Association for data collection and from the developer of the instrument for using NMCS^©,^.^33^ All potential participants received information about the study and had the opportunity to obtain additional information. Confidentiality and anonymity were guaranteed and participation was voluntary. Returning the completed questionnaire was considered as consent to participate.^54,55^

## Findings

### Potential whistle-blowers

A total of 454 nurses (31%) returned the questionnaire with a narrative and completed NMCS©. Most of the PW-Bs were female (95%) with a mean age of 47 years and mean length of work experience of nearly 20 years. The majority were registered nurses (90%) with a polytechnic degree (53%) and were currently working as employees (63%). PW-Bs mostly viewed their own personality as between an introvert and extrovert (50%). Other personality and social responsibility related variables were measured using a VAS scale 0–100. Out of personality related variables, PW-Bs assessed the level of respecting their own profession as the highest (mean 85.2) and allowing others to influence their own opinions (44.8) as the lowest. In addition, the level of social responsibility for patients or their next of kin was assessed as the highest (84.6). (Table 1.) The majority of the PW-Bs would address the whistleblowing act inside the organisation (98%) and only two percent externally outside the organisation.

The majority of the PW-Bs would address the whistleblowing act inside the organisation (98%) and only two percent externally, outside the organisation. In addition, they considered it would be easiest to act morally courageously when facing co-workers (mean level 4.25, SD 0.80) and the easiest way would be open discussion among the working team members (mean level 4.16, SD 0.83) (Table 2).

### Moral courage of the potential whistle-blowers

The mean level of the PW-Bs’ moral courage was 4.34 (SD 0.47). Of sum variables, *Compassion and true presence* was assessed as the highest with a mean of 4.55 (SD 0.46) while *Commitment to good care* was the lowest, with the mean of 4.21 (0.56). Mean of their self-assessed overall moral courage (VAS 1–10) was 8.55 (0.84). (Table 3.)

### Potential whistle-blowers’ background variables associated with moral courage

Out of the PW-Bs’ background variables, socio-demographics, education, work, personality and social responsibility related variables were statistically significantly associated with their self-assessed level of moral courage (Tables 4 and 5). PW-Bs with other degrees showed significantly higher moral integrity than public health nurses, midwives and nurse paramedics (*p* = 0.032). In addition, those with the highest degrees assessed themselves as more morally courageous than students (*p* = 0.031) and those having a polytechnic degree (*p* = 0.034) and showed higher moral responsibility than those with a vocational (*p* = 0.048) or polytechnic (*p* = 0.039) degree. (Table 4.)

**Table 5.** Correlations between background variables and moral courage and its dimensions.

Background variable	Total *p*-value r	CTP *p*-value r	MR *p*-value r	MI *p*-value r	CGC *p*-value r	Overall *p*-value r
Age	0.007* 0.15	—	—	0.001** 0.17	0.007** 0.16	<0.001** 0.28
Work experience	0.02* 0.13	—	—	0.003** 0.16	0.01* 0.14	<0.001** 0.22
Respecting own profession	<0.001** 0.35	<0.001** 0.31	<0.001** 0.28	<0.001** 0.33	<0.001** 0.32	<0.001** 0.27
Self-esteem	<0.001** 0.3	<0.001** 0.22	<0.001** 0.23	<0.001** 0.3	<0.001** 0.3	<0.001** 0.29
Internal locus of control	<0.001** 0.3	<0.001** 0.25	<0.001** 0.25	<0.001** 0.28	<0.001** 0.28	<0.001** 0.24
SR patient/next of kin	<0.001** 0.36	<0.001** 0.35	<0.001** 0.29	<0.001** 0.32	<0.001** 0.3	<0.001** 0.32
SR co-workers	<0.001** 0.29	<0.001** 0.23	<0.001** 0.27	<0.001** 0.3	<0.001** 0.24	<0.001** 0.2
SR work community well-being	0.000** 0.4	0.000** 0.31	<0.001** 0.33	<0.001** 0.4	<0.001** 0.36	<0.001** 0.29
Allowing others to influence own opinions	<0.001** −0.25	<0.001** −0.2	<0.001** −0.23	<0.001** −0.24	<0.001** −0.21	<0.001** −0.19
E co-worker	<0.001** 0.36	<0.001** 0.27	<0.001** 0.33	<0.001** 0.36	<0.001** 0.32	<0.001** 0.33
E colleague	0.000** 0.41	<0.001** 0.29	<0.001** 0.35	0.000** 0.43	<0.001** 0.37	<0.001** 0.36
E health care manager	<0.001** 0.36	<0.001** 0.21	<0.001** 0.34	0.000** 0.39	<0.001** 0.32	<0.001** 0.31
E physician	<0.001** 0.37	<0.001** 0.24	<0.001** 0.32	0.000** 0.4	<0.001** 0.32	<0.001** 0.35
E organization	<0.001** 0.38	<0.001** 0.22	<0.001** 0.34	0.000** 0.41	<0.001** 0.35	<0.001** 0.36
E party outside the organization	0.000** 0.4	<0.001** 0.28	<0.001** 0.36	0.000** 0.39	<0.001** 0.35	<0.001** 0.34
E patient	0.000** 0.42	<0.001** 0.3	<0.001** 0.38	0.000** 0.4	<0.001** 0.37	<0.001** 0.38
E patient’s next of kin	0.000** 0.42	<0.001** 0.31	<0.001** 0.36	0.000** 0.39	<0.001** 0.38	<0.001** 0.39
E BPD	0.000** 0.47	<0.001** 0.33	<0.001** 0.39	0.000** 0.47	0.000** 0.44	<0.001** 0.31
E FN	<0.001** 0.4	<0.001** 0.32	<0.001** 0.33	<0.001** 0.38	<0.001** 0.37	<0.001** 0.26
E EWB	<0.001** 0.27	<0.001** 0.18	<0.001** 0.28	<0.001** 0.24	<0.001** 0.21	<0.001** 0.26
F using guidelines	<0.001** 0.21	<0.001** 0.18	<0.001** 0.14	<0.001** 0.21	<0.001** 0.24	<0.001** 0.22
F of situations	<0.001** 0.26	<0.001** 0.19	<0.001** 0.28	<0.001** 0.26	<0.001** 0.2	<0.001** 0.22

Statistically significant *p*-values are indicated in the table: *p < 0.05; **p < 0.01.

r: Spearman’s correlation coefficient.

Dimensions of moral courage CTP: Compassion and true presence; MR: Moral responsibility; MI: Moral integrity; CGC: Commitment to good care; SR: Social responsibility; E: Easiness to, BPD: bring the problem into discussion, FN: file a notification with the organization, EWB: external whistleblowing, F: frequency.

Within all the dimensions of moral courage, those PW-Bs working as health care managers showed significantly higher level of moral courage than employees. In addition, overall moral courage was assessed as highest by those who were working in other roles than as employees. PW-Bs who considered their personality closer to extrovert showed a significantly higher level of moral courage in all the dimensions. (Table 4.)

The highest positive correlations were between ease of bringing the problem up for discussion and total moral courage and moral integrity (both r = 0.47). The highest correlations concerning personality related variables were between respecting one’s own profession and total moral courage (r = 0.35). In addition, the highest correlations concerning social responsibility were between the well-being of the work community and total moral courage and moral integrity (both r = 0.4). Negative correlations were identified between allowing others to influence one’s own opinions and moral courage. The highest correlation was seen between ease of acting morally courageously when facing colleague and moral integrity (r = 0.43). (Table 5.)

## Discussion

This study produced novel evidence by identifying potential whistle-blowers in health care, their moral courage and background variables associated with it for the use of future decision-making and to support and encourage healthcare professionals in their whistleblowing. The PW-Bs assessed their moral courage as rather high, slightly higher than previous studies describing nurses’ moral courage.^33,34,36^ The results are encouraging, as it is suggested that high level of moral courage entails higher probability of whistleblowing.^47^ However, it needs to be acknowledged that there is a bias for socially desirable responses when using the video-vignette method^57^ and self-assessment instrument.^58^

In this study, the PW-B was identified. The PW-B represents an average nurse on a national level in terms of socio-demographics, education and work related variables,^59^ increasing the external validity of the study.^52^ The findings indicate that relying often on ethical guidelines^60^ and previous experiences of encountering situations requiring moral courage could enhance moral decision-making and acting morally courageously if facing acts of wrongdoing such as the one presented in the video vignette. In addition, relying on ethical guidelines entails PW-Bs’ professionalism as healthcare professionals.^61^

PW-Bs considered their personality type to be between an introvert and extrovert, assessed their self-esteem as quite good, and allowed others to influence their own opinions to a moderate degree. These are individual personality variables which they probably had even before entering the health care profession. Furthermore, PW-Bs assessed their internal locus of control on an average level, indicating that they do not believe in being in total control of the events around them. One plausible explanation for this could be the hierarchical nature of health care.^32,62^ The findings of this study are encouraging as previous studies suggest that high extraversion,^63^ adequate self-esteem and high level of internal locus of control predict prosocial behaviour in the form of whistleblowing.^18^

The findings indicate that PW-Bs have a strong role and group identity as nurses as they respected their own profession highly and described high levels of social responsibility for others. Responsibility for patients was assessed as the highest, which is part of being a nurse, nurses’ accountability, and a fundamental issue of being the patient’s advocate.^8,61,64^ It is encouraging that whistleblowing is potentially increased by both a strong role and group identity.^61^

According to the results of this study, PW-Bs assessed their overall moral courage rather high in all the dimensions of the NMCS©, which is consistent with previous research among nurses. However, in this study, moral courage was assessed higher than in previous studies.^33,34,36^ The difference might be caused by the fact that here, participants were potential whistle-blowers and therefore, they could be more morally courageous. In addition, moral courage, as a highly appreciated human characteristic, may increase socially desirable responses due to individuals’ desire to present themselves in a socially or professionally acceptable light.

Of the dimensions of moral courage, compassion and true presence was assessed as the highest, with similar results presented in previous research.^33,34^ The results suggest that PW-Bs possess empathy and that being patients’ advocate is a crucial part of their professional role and important for them. Furthermore, being patients’ advocate is one of the main facilitators for whistleblowing in health care.^8,64^ The dimension of commitment to good care was assessed the lowest, which is both consistent^34^ and inconsistent^33^ with previous studies, with the latter reporting moral responsibility as the lowest dimension. However, even the lowest dimension was assessed on a quite high level, indicating courage to act in situations where wrongdoing occurs or good care is threatened for other reasons.

The findings of this study confirm particularly the associations between PW-Bs’ moral courage and education related variables as well as the work role as manager, which is usually achieved through education. Consistent with the Aristotelian view, virtues such as moral courage can be developed through practice and education.^31^ In addition, the findings are supported by earlier suggestions that moral courage as part of ethical and professional competence could be developed and strengthened with education.^33,34,65^ It is encouraging that the highest degree was associated with moral courage, as earlier literature indicates it as one of the predictors of whistleblowing.^66^ Inconsistent findings report that the highest degree was not associated with moral courage.^36^ Parallel results on the current work role were presented as managers assessed their level of moral courage higher than employees.^36^ The findings indicate that higher position in professional hierarchy contributes to moral courage, which is supported by earlier literature.^32,41^ In addition, managers have responsibility to act as courageous examples and support employees in their moral courage and their whistleblowing.^65^

Personality related variables were associated with moral courage, implying that morally courageous PW-Bs possess certain personality traits which they assessed at a rather high-level. However, further research is needed to compare whether there is a difference between healthcare professionals’ and whistle-blowers’ moral courage and their personality traits or whether these traits are something that all the healthcare professionals possess, as it is suggested that individuals who have chosen the health care profession are usually willing to help others and to act morally courageously and put themselves at risk for the benefit of others.^67^ Nevertheless, the findings are encouraging as the personality variables extroversion, self-esteem, locus of control and social responsibility support moral courage and its manifestation as a form of whistleblowing.^18,63,68–70^

The COVID-19 pandemic has emphasised healthcare professionals’ need for moral courage and acting morally courageously as they have exposed themselves to a severe risk of a contagious and potentially deadly virus for the goal of doing good to others. Moral courage and courageous action are not only dependent on individual variables, but also on situational and contextual factors^25^ such as the pandemic, the type of observed wrongdoing,^6^ ethical climate,^71^ management, organisational culture,^9,65,72^ support received from others, and hierarchical power structures in health care.^17,73^ To get an extensive and broader understanding, these variables and their associations with moral courage and whistleblowing as a courageous action require further research.

### Methodological considerations and limitations

There are some methodological considerations and limitations in this study. Firstly, a simulated video vignette method was used, and one of the theoretical limitations described is the gap between social reality and the vignette.^57^ However, there is evidence that vignettes may predict participants responding similarly to real-life situations as to simulated vignettes.^49^ In addition, vignettes enable distancing participants from potentially difficult and sensitive research topics,^50^ such as whistleblowing. Video vignettes are considered more realistic than written ones, enabling observers to immerse themselves in the situation.^57^ Therefore, the video vignette method was chosen, and the video vignette was pre-tested. Secondly, the sample was recruited from one national nurses’ association with a relatively low response rate (31%), although it included nurses who formed the target population of this study. Based on the statistical power analysis, the number of participants was adequate. In addition, the participants were working in various organisations, representing an average nurse. Thirdly, data were collected from one country with a culturally specific health care system and prevailing social norms. However, nurses share globally universal values and similar ideology with other healthcare professionals. Fourthly, the reliability and validity of the NMCS^©^ has been proven and it has been developed specifically for the nursing context.^33,34^ Lastly, a social desirability bias exists as the video vignette method was used^57^ and moral courage was measured using a self-assessment instrument.^58^ However, the vignette method potentially reduces socially desirable responses.^50^

### Implications and further research

This and previous studies^33,34,36^ indicate that nurses as healthcare professionals, assess their moral courage at a rather high level. However, earlier literature suggests that only around half of the healthcare professionals observing wrongdoing actually blow the whistle.^6^ Therefore, it should be considered whether the barrier for whistleblowing is lack of courage^64^ or lack of knowledge of the manifestation of moral courage in different ways such as whistleblowing. Ethics training and ethics interventions could be beneficial for effective manifestation of moral courage as moral courage can be strengthened with education.^72,74^ Further research is required on whether the low whistleblowing rates and performing whistleblowing acts in real-life are associated with moral courage. In addition, research is needed to explore whether moral courage and the background variables identified here predict actual whistleblowing. Exploring social, situational, and individual predictors for whistleblowing is also needed in the future. For generalising the results of this study, international research needs to be conducted in different countries, cultures, and health care services.

There is a growing interest in developing legislation for whistle-blower protection^75^ as whistleblowing can be seen as an effective tool for developing the organisation.^9,76^ Therefore, organisational structures, processes and management should support whistleblowing and create whistleblowing opportunities by providing whistleblowing channels. In addition, employees should be encouraged to blow the whistle.^77^ Moral courage and whistleblowing are associated with professional and organisational hierarchy.^6,20^ It could be beneficial to reduce professional hierarchy in health care organisations with different management styles such as involved management. Furthermore, the workplace culture should be open and transparent so that it is easy to raise concerns and speak up about wrongdoing.

## Conclusion

Potential whistle-blowers seem to be quite morally courageous actors when responding to a simulated wrongdoing. The identification of potential whistle-blowers, their moral courage and the variables associated with it offers opportunities for developing interventions, practices, and education to support and encourage healthcare professionals in their whistleblowing. Research could focus on developing a theoretical construction aimed at eventually increasing whistleblowing and decreasing and preventing wrongdoing.
